# Do Public Schools Provide Optimal Support for Children With Diabetes?

**Published:** 2007-06-15

**Authors:** Dustin Melton, Jessica Henderson

**Affiliations:** Child and Adult Care Food Program Specialist, Arizona Department of Education, Phoenix, Ariz 85007; Associate Professor, Western Oregon University, Monmouth, Ore 97361

## To the Editor:

The obesity epidemic among children has received considerable attention in the media, partly in response to two important reports by the Institute of Medicine on the topic (1,2). The parallel rise in diabetes mellitus among children is an equally important topic but is less discussed, even though diabetes has become one of the most common chronic diseases among youth. The estimated prevalence of type 1 or type 2 diabetes mellitus among U.S. children or youth aged 17 years or younger was 3.2 per 1,000 (which means that there were about 229,240 children or youth aged <18 years with diabetes in the United States) in 2003–2004 (3). The public schools are important locations for secondary prevention interventions to help these children and youth minimize their risk for complications associated with diabetes. We investigated the diabetes knowledge and preparedness among members of the faculty and staff of schools in Oregon, as well as barriers to school support for students with diabetes.

We asked administrators and staff listed in the Public School Directory on the Oregon Department of Education Web site in 2005 to complete the *School Diabetes Survey* developed by Lewis et al (4). The 26-item survey examined in-school support for students with diabetes, including the diabetes-related knowledge and training of teachers and staff and the schools' policies and procedures regarding diabetes.

The 135 schools that responded to the survey represented 19% of the K–12 public schools in the state with an e-mail address in the directory of the Oregon Department of Education, and the student population of the 135 schools represented 11% of the total student population in Oregon. Of the 126 schools that indicated their school level, 48% were elementary schools only, 15% were middle schools only, 21% were high schools only, and the rest were mixed levels. The population of the schools ranged from 50 to 2404 students.

The number of students identified with diabetes in each school ranged from none to 18. One of every four schools reported that they had no students with diabetes. About 80% of the schools indicated that teachers or school staff received training about diabetes. Likewise, 80% stated that they had a school diabetes policy in place. This finding means at least one in five schools either did not have a policy regarding the management of students with diabetes, did not have a person trained to understand the needs of students with diabetes, or both (Table 1).

Almost all schools (94%) responded that they had a policy that allowed students to measure their own blood glucose at school; only two (one high school and one elementary school) responded that they did not. Most schools (70%) reported believing that the responsibility for providing glucose-containing food or beverages or both for students with diabetes resides with the parents. Three of every four schools had an accessible refrigerator to house glucose-containing foods. Only two thirds of schools had a full-time staff person who was trained to administer insulin injections. Nearly half of the schools perceived that the main barrier to providing optimum support for students with diabetes was the lack of a nurse on staff each day (Table 2). Lack of teacher and staff training and lack of funding were also cited as being the main barrier.

The results from our study were similar to those from a study of Lewis et al of 222 schools in the East, which showed that 17% of schools did not have a staff member with training about diabetes and that 10% did not have a diabetes management policy (4). Absence of a nurse on site daily was a common finding in that study also, indicating that these issues regarding the capacity of schools to assist students with diabetes most likely exist in other states.

The National Association of School Nurses recommends that schools should have one school nurse for every 750 students (5). Unfortunately, the national ratio is just one for every 1350 children. This means that many children receive care from a secretary, teacher, or counselor. Under these conditions, it is critical that schools redouble their efforts to make sure they have a safe and secure process for providing optimum support for students with diabetes.

One educator in this study described the lack of support provided to students with diabetes this way: "even though diabetes is serious, it does not receive the same publicity and criticism as does state testing, school report cards, etc. That's what makes the news and gets attention. Diabetes, along with all the other ailments, is serious, but get us (especially small rural schools) medical help." The findings of this study indicate that at least one in five Oregon schools is lacking what it needs to provide a healthy environment for students with diabetes: either a nurse on staff during all school hours, diabetes training for teachers and staff, or both.

## Figures and Tables

**Table 1:** Percentage of Schools Surveyed That Reported Having a Policy Regarding the Management of Students with Diabetes, by School Level, Oregon, 2005

School Level (n)^a^	Policy	No Policy	Don't Know
Elementary (61)	79%	18%	3%
Elementary/Middle (6)	67%	33%	
Middle (19)	95%	5%	
Middle/High (5)	80%	20%	
High School (28)	82%	18%	
Elementary/Middle/High (7)	57%	43%	

a Of 135 schools surveyed, only 126 provided information on school level.

**Table 2:** Main Perceived Barrier to Providing Support to Students With Diabetes in Oregon, 2005

Barrier	% of Schools^a^ (n = 113 )
Registered nurse not on staff every day	48%
Diabetes not perceived as a problem	31%
Lack of diabetes training for teachers and/or staff	22%
Lack of funding	15%
Lack of parental involvement	1%

a Sum of percentages exceeds 100% because some schools reported more than one barrier.
